# In vivo magnetic resonance imaging study of the hip joint capsule in the flexion abduction external rotation position

**DOI:** 10.1038/s41598-022-10718-7

**Published:** 2022-04-22

**Authors:** Masahiro Tsutsumi, Isao Yamaguchi, Akimoto Nimura, Hajime Utsunomiya, Keiichi Akita, Shintarou Kudo

**Affiliations:** 1 Inclusive Medical Science Research Institute, Morinomiya University of Medical Sciences, 1-26-16 Nankokita, Suminoe-ku, Osaka city, Osaka, 559-8611 Japan; 2 Department of Clinical Anatomy, Graduate School of Medical and Dental Sciences, Tokyo Medical and Dental University, Tokyo, Japan; 3 Department of Radiological Science, Faculty of Health Science, Morinomiya University of Medical Sciences, Osaka, Japan; 4 Department of Functional Joint Anatomy, Graduate School of Medical and Dental Sciences, Tokyo Medical and Dental University, Tokyo, Japan; 5 Tokyo Sports and Orthopaedic Clinic, Tokyo, Japan

**Keywords:** Musculoskeletal system, Orthopaedics

## Abstract

Although the flexion abduction external rotation (FABER) test is a useful hip provocation test, hip soft tissue characteristics in the FABER position remain unclear. This study investigated the *in-vivo* joint capsule characteristics, including its articular cavity area and relation to the fat pad surrounded by the joint capsule and pericapsular muscles, in the FABER position using magnetic resonance imaging. Thirteen hips from 13 healthy volunteers were analyzed. The images were obtained, with the participant hips at 15°-extension, 45°-flexion, and in the FABER position, to analyze the articular cavity size and fat pad and calculate these ratios to size of the femoral neck. The articular cavity area and its ratio to the femoral neck were significantly greatest in the FABER position, followed by those in the hip flexion and extension. Additionally, the area of the fat pad in the inter-pericapsular muscle space and its ratio to the femoral neck in the FABER position were significantly larger than those in the hip flexion and, as a tendency, larger than those in hip extension. To the best of our knowledge, this is the first *in-vivo* study to show the interrelationship among the joint capsule, pericapsular muscles, and fat pad in the FABER position.

## Introduction

The flexion abduction external rotation (FABER) test, also known as Patrick’s test, is commonly used as a physical examination modality to assess hip, lumbar spine, or sacro-iliac joint pathology^1^. The FABER assessment is performed with the individual in a supine position, with the thigh flexed and the ankle placed on the opposite extended knee (FABER position)^2^. Several studies have shown the utility of the FABER test as a hip provocation test, especially for detecting femoroacetabular impingement and acetabular labrum tears^3–5^. For an appropriate hip pathology diagnosis, it is vital to understand the hip anatomical structure, both of bone and soft tissues, subjected to mechanical stress in the FABER position.

Previous studies have reported in vivo bony alignment at the FABER position using computed tomography or magnetic resonance (MR) imaging^6–9^. In contrast, soft tissue characteristics in the FABER position remain unclear; it has only been speculated that a decrease in hip joint range of motion during a FABER test may indicate either joint capsule tightness or psoas spasm^3^. A recent anatomical study suggested that the joint capsule can intrude toward the neck of the femur with hip extension, thereby forming the zona orbicularis, and the articular cavity is three-dimensionally narrowed by this protrusion^10^. Therefore, morphological knowledge of the in vivo joint capsule morphology in the FABER position, especially about the articular cavity area, may also aid in making an appropriate hip pathology diagnosis using the FABER test. Additionally, a recent arthroscopic study suggested that the degenerative fat pad (such as blood vessel-rich or fibrous fat pad or replaced by fibrous scar tissue) on the anterior inferior iliac spine—between the proximal rectus femoris and anterior joint capsule—is closely related to a FABER test-provoked anterior hip pain^11^. This fat pad is surrounded by a joint capsule and pericapsular muscles^12^; hence, an analysis of the joint capsule itself and its relation to the fat pad and pericapsular muscles may be vital in understanding the morphological characteristics of the hip in the FABER position.

The aim of this study was to elucidate the in vivo morphological characteristics of the joint capsule, including its articular cavity area and its relation to the fat pad, in the FABER position using MR imaging, by comparison with those in hip flexion and extension in which the characteristics of loose/tight joint capsule may be shown.

## Methods

### Participants

Thirteen hips from 13 healthy volunteers (13 men; participant characteristics are shown in Table 1) were investigated. Women were excluded due to gender differences in the stiffness of connective tissues such as ligaments and tendons in many joints; moreover, the menstrual cycle effects on joint laxity in women are still being debated^13–16^. The study design was approved by the ethics committee of Morinomiya University of Medical Sciences (#2021–022), and all procedures were performed in accordance with the Declaration of Helsinki (last modified in 2013) and the Japanese guideline entitled, “Ethical Guidelines for Medical and Health Research Involving Human Subjects.” All participants provided written informed consent.

**Table 1:** Participant characteristics.

	*n* = 13
Age (years)	22.5 ± 3.4
Weight (kg)	66.9 ± 10.3
Height (m)	1.72 ± 0.06
BMI (kg/m^2^)	22.5 ± 2.5
**Hip range of motion (°)**	
Flexion	106.3 ± 8.9
Extension	22.5 ± 3.2
Adduction	17.5 ± 6.6
Abduction	35.8 ± 5.7
Internal rotation	24.6 ± 7.8
External rotation	43.8 ± 6.8
**FABER**	
Height (cm)	11.0 ± 2.3
Height-normalized thigh length	0.29 ± 0.07

Data are expressed as mean ± standard deviation.

BMI, body mass index; FABER, flexion abduction external rotation.

We included participants with no history of hip surgery. Participant hip passive range of motion in supine was evaluated to confirm the absence of an apparent range of motion limitation or pain in the hip; further, the FABER test was performed while measuring the perpendicular height from the lateral femoral epicondyle to the bed using a ruler with 1-mm increments^17^. We also divided this height by the thigh length between the greater trochanter and lateral femoral epicondyle to calculate the height-normalized thigh length of the FABER test^17^. Measurement data are shown in Table 1; no participant showed an apparent range of motion limitation or pain in the hip. Therefore, all 13 participants were assigned for MR imaging analysis.

### MR imaging analysis

**Figure 1:**
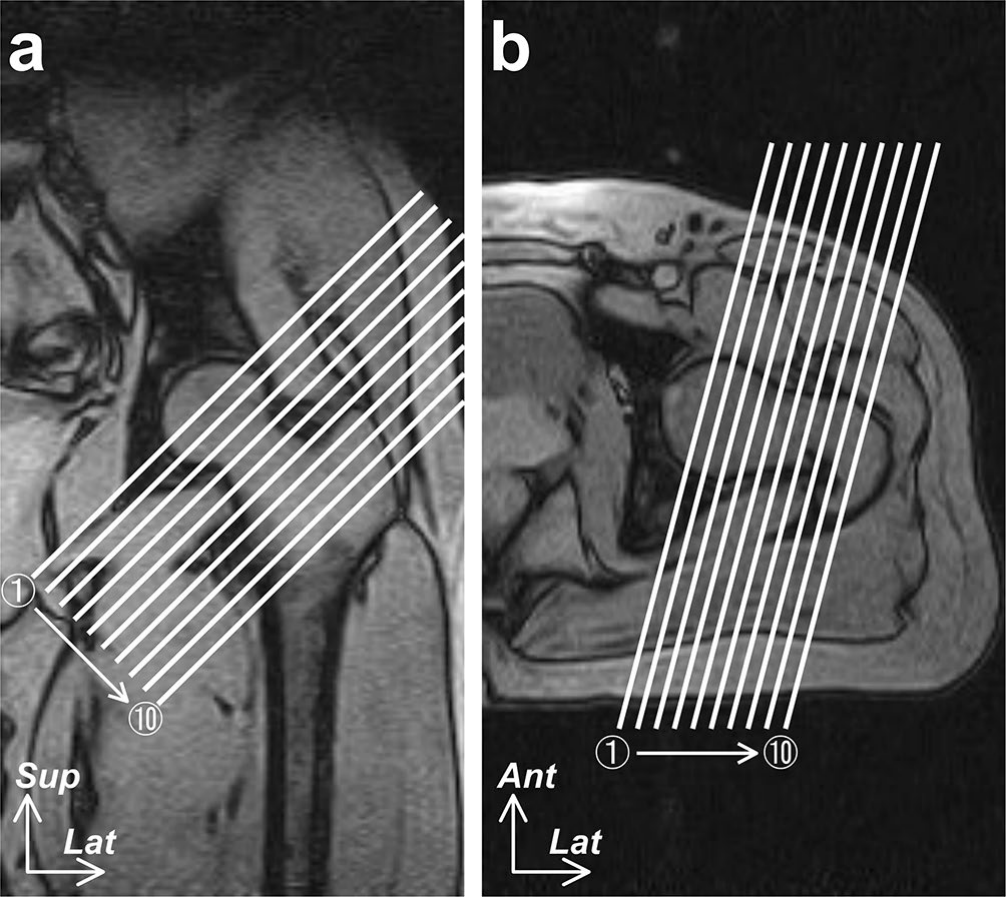
Slice location and imaging direction of the magnetic resonance imaging. Coronal (**a**) and horizontal (**b**) sections of the left hip in extension. The locations and directions of the slices are indicated by the white lines. *Ant* = anterior, *Lat* = lateral, and *Sup* = superior.

Joint capsule imaging was performed with a 0.3 T MR imaging system (AIRIS Vento; Hitachi, Ltd., Tokyo, Japan) using a quadrature detection body coil (MR-QFC-102AN; Hitachi, Ltd., Tokyo, Japan). Because the articular cavity is three-dimensionally narrowed by the joint capsule protrusion to the neck of the femur, the cross-sectional cavity area, slicing perpendicular to the neck axis, at the narrowest level of the neck can be used to quantitatively evaluate the dynamic changes in the joint capsule as a whole^10^. Therefore, images showing the axial plane perpendicular to the axis of the neck of the femur were acquired as T_2_-weighted (T2W) images using a fast spin-echo sequence (Fig. 1), ranging from the head of the femur to the intertrochanteric line or crest (10 slices of MR images). The MR parameters were as follows: repetition time/echo time, 4000/120 ms; flip angle, 90°; echo train length, 11; 256 × 192 matrix; 25-cm field of view; 5.0-mm sections; 1.0-mm interval; and acquisition time, 9 min 8 s per position. Participants were placed in a supine position, and each hip was maintained in the center of a magnet bore at all positions. Images were obtained with the left hip in three different positions, using previously described methods^6,7^: 15° of hip extension, 45° of hip flexion, and the FABER position; these images were then compared to clarify the joint capsule characteristics in the FABER position.

The MR image of the level at which the neck of the femur was the narrowest was selected from 10 slices of MR images per position, and the areas of the articular cavity and fat pad were measured using ImageJ software (version 1.52; National Institutes of Health, Bethesda, MD, USA) and compared among the three positions. The area of the articular cavity was defined as the area occupied by the synovial fluid (with high-signal intensity on T2W images) and surrounded by the joint capsule (with low-signal intensity). To verify that its area at the femoral neck narrowest level represented the entire volume of the articular cavity, the sum of all 10 slices articular cavity area multiplied by the slice thickness (5 mm) was defined as the entire articular cavity volume and the correlation between the area at the femoral neck narrowest level and volume was analyzed in three hip positions of 13 subjects, a total of 39 cases. The area of the fat pad was defined as the high-signal intensity area, located deep to the rectus femoris, medial to the gluteus minimus, lateral to the iliopsoas, and superficial to the anterior joint capsule^12^. Moreover, the cross-sectional area of the neck of the femur was measured, and the ratio of the articular cavity and fat pad area to the neck of the femur area was calculated.

Each region of interest was semi-automatically determined using “Wand tool” of ImageJ by the following method. We selected and clicked inside the region of interest, and the pixel value of the clicked area was automatically extracted (Supplementary Figure S1a). Next, we set the tolerance value, and the object outline was automatically selected under the condition that all pixel values in that area were in the range from “clicked pixel value − tolerance” to “clicked pixel value + tolerance” (Supplementary Fig. S1b). The tolerance value was determined to ensure that no region beyond the outline of the object was selected. Using this method, all regions of interest—the femoral neck, articular cavity, and fat pad—were selected (Supplementary Fig. S1c). All measurements were performed twice by a single observer, and the average of two measurements was recorded for statistical analysis. Intraclass correlation coefficients (ICCs) were calculated to determine the intra-rater reliability of each measured value.

### Evaluation metrics of the selected slices

**Figure 2:**
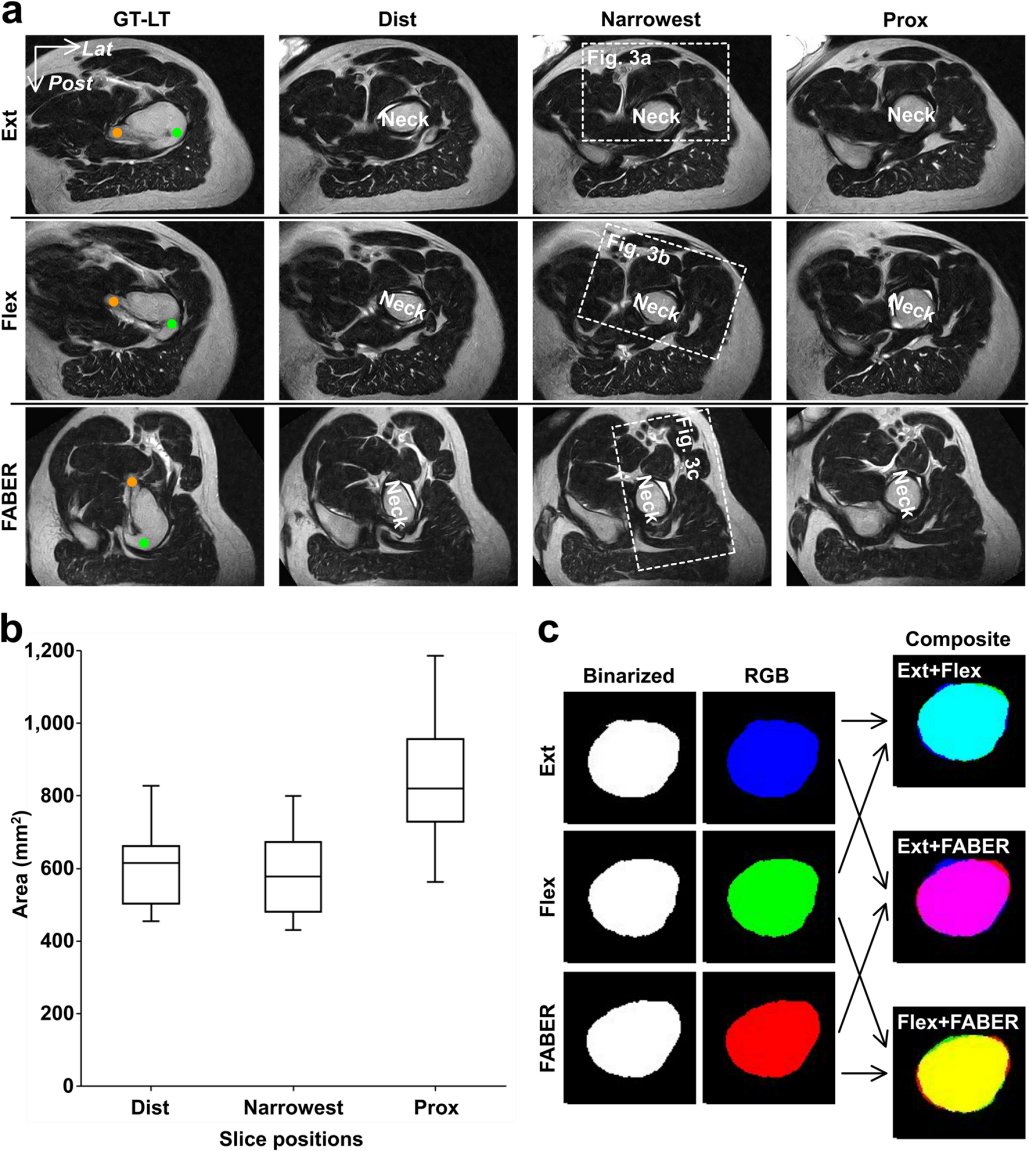
Evaluation methods of the selected slices. (**a**) Axial sections perpendicular to the femoral neck (Neck) axis in the hip extension (Ext, upper row), flexion (Flex, middle row), and FABER position (FABER, lower row), at the level of the greater and lesser trochanter (GT-LT, first column from the left), narrowest level of the femoral neck (Narrowest, third column) and its distal/proximal adjacent slice level (Distal and Prox, second and fourth column, respectively). Green and orange circles at the GT-LT column indicate the greater and lesser trochanter, respectively. The boxed regions at the Narrowest column indicate the orientation of Fig. 3. (**b**) Box plots show the cross-sectional area of the femoral neck at the narrowest level (Narrowest) and its distal/proximal adjacent slice level (Distal and Prox, respectively). The height of the boxplot represents the interquartile range (IQR), and the black horizontal line inside the box represents the median. The lower and upper whiskers extend to the lowest and highest values within 1.5 IQR of the first and third quartiles, respectively. (**c**) Analyzed images of the narrowest femoral neck in the three hip positions. First column from the left indicates the Binarized images of the femoral neck (Binarized); second, those colored binarized image (RGB); and third, a composite image (Composite). Upper letters in the Composite column indicates the hip position combination (e.g., “Ext + Flex” means the composite images of the blue image in hip extension and green image in hip flexion). *Lat* = lateral and *Post* = posterior.

To verify that the MR image of the level at which the femoral neck was the narrowest was selected, the cross-sectional areas of the femoral neck were also measured at the proximal/distal adjacent slices (Fig. 2a). The femoral neck area at the selected slices (median [interquartile range, IQR]: 577.0 [478.4–671.8] mm^2^) was narrower than those at the proximal (817.9 [726.9–954.1] mm^2^) and distal adjacent slices (612.6 [501.4–659.7] mm^2^) (Fig. 2b).

Additionally, to quantitatively measure whether the MR images taken in the three hip positions were selected at the same level of the femoral neck per participant, we calculated the Dice similarity coefficient (DSC), a validation metric for the quantitative evaluation of the two samples similarity^18^. In general, given two sets, X and Y, the DSC is defined as follows:

1 $${\text{DSC }} = { 2}\left| {{\text{X}} \cap {\text{Y}}} \right| \, / \, \left( {\left| {\text{X}} \right| + |{\text{Y}}|} \right)$$

First, using ImageJ, the selected slices per participant were rotated to align those orientations based on the greater and lesser trochanter location, and those binarized images of the femoral neck were created in three hip positions (Fig. 2c; “Binarized” column). Second, the color of the binarized image in the hip extension was changed to blue, those in the hip flexion to green and those in the FABER position to red (Fig. 2c; “RGB” column). Third, two of the three images (e.g. those in hip extension and flexion) were combined to create a composite image, and calculate the overlapped area (Fig. 2c; “Composite” column). Finally, the DSC of two images were calculated based on the formula (1) as follows:

2 $$\begin{aligned} {\text{DSC }} = { 2}*{\text{overlapped }} & {\text{area of the composite image}} \\ /{\text{ Sum of the area of the two binarized images}} \\ \end{aligned}$$

The DSCs between the MR images in the hip extension and flexion, in the hip extension and FABER position, and in the hip flexion and FABER position were 0.95 (range, 0.92–0.98), 0.95 (range, 0.91–0.99), and 0.94 (range, 0.91–0.97), respectively. A DSC value ≥ 0.70 generally indicates excellent agreement^19^. All DSCs were ≥ 0.91.

### Statistical analyses

Statistical tests were performed using SPSS (version 27.0; IBM Corp, Armonk, NY, USA). Statistical comparisons of the articular cavity and fat pad areas, among the abovementioned three hip positions, were performed using the Friedman test and its significance level was set at *p* < 0.05. Additionally, paired comparisons of the articular cavity and fat pad areas between hip extension and flexion, extension and FABER, and flexion and FABER, were performed using the Wilcoxon signed-rank test with Bonferroni correction and its significance level was set at *p* < 0.017. Data are presented as median and interquartile range (IQR).

**Figure 3:**
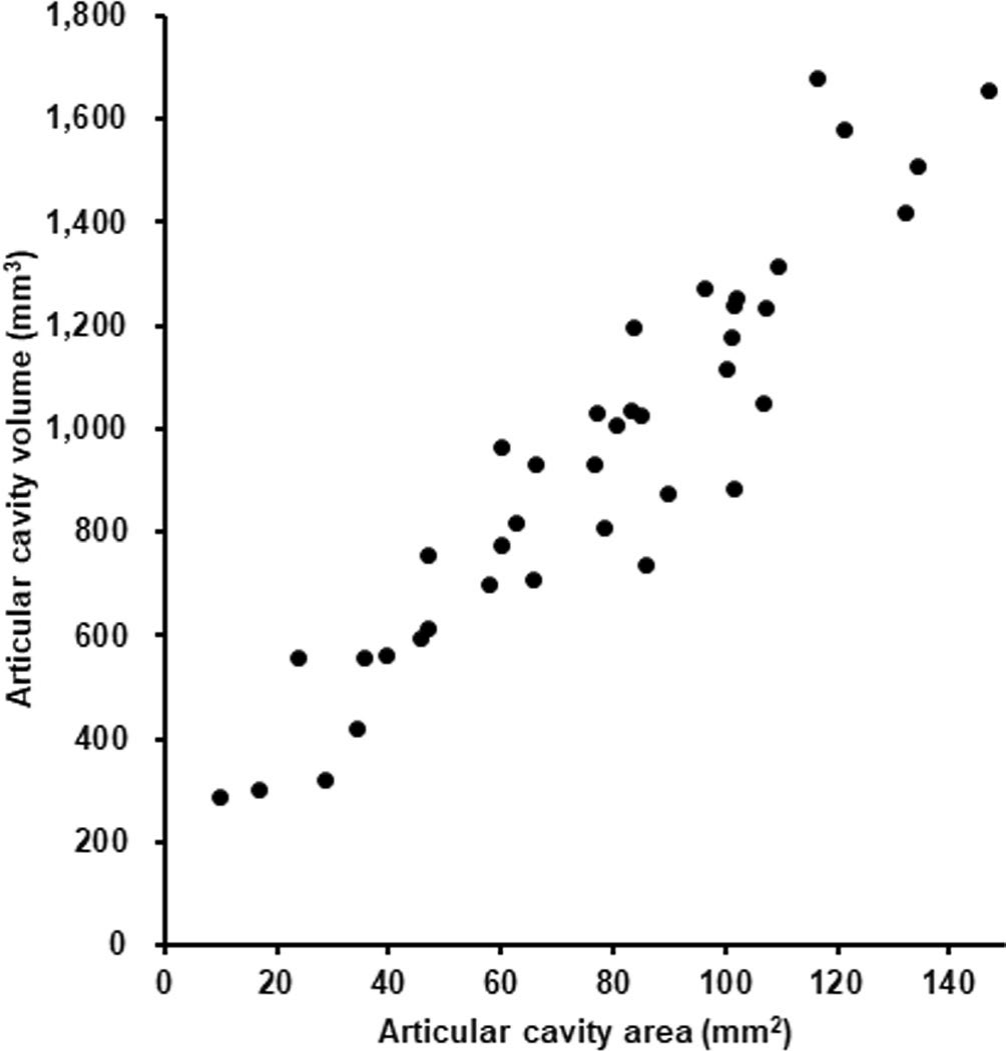
Correlation between the articular cavity area at the narrowest level of the femoral neck and the entire articular cavity volume. The area at the narrowest level of the femoral neck was significantly and highly correlated with the entire articular cavity volume (Spearman correlation; rho = 0.93, *p* < 0.001).

Statistical correlation between the articular cavity area at the narrowest level of the femoral neck and the entire articular cavity volume was examined using Spearman correlation, with a significance level of *p* < 0.05. The area at the narrowest level of the femoral neck was found to be significantly and highly correlated with the entire articular cavity volume (Fig. 3; rho = 0.93, *p* < 0.001). Therefore, the articular cavity area at the narrowest level of the femoral neck was validated to represent the value of the entire articular cavity volume.

The ICCs of the neck of the femur, articular cavity and fat pad area measurement were 0.98 [95% CI 0.96–0.98], 0.98 [95% CI 0.97–0.99] and 0.997 [95% CI 0.994–0.998], respectively. An ICC score ≥ 0.75 was considered to indicate an excellent agreement^20^. All ICCs were ≥ 0.96 (range, 0.96–0.998), indicating excellent agreement.

## Results

**Figure 4:**
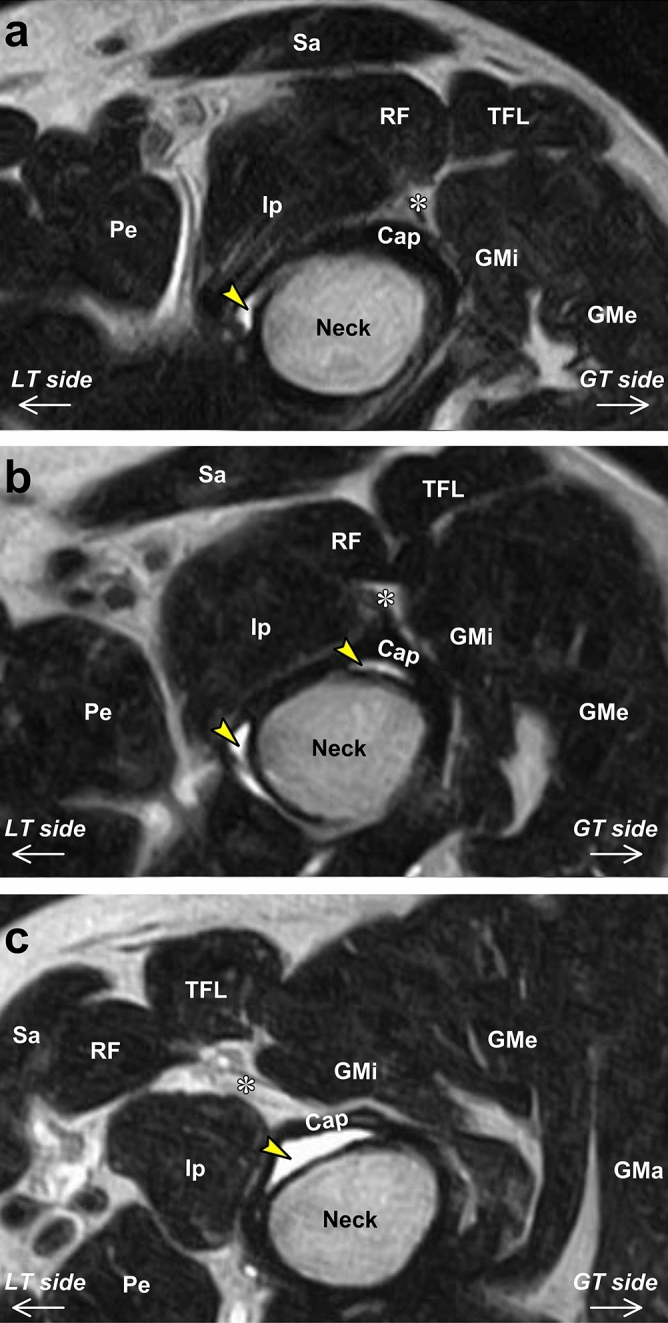
Axial sections perpendicular to the axis of the neck of the femur in three hip positions. Axial sections at the narrowest level of the neck of the femur (neck) in the hip flexion (**a**), hip extension (**b**), and FABER (**c**) positions. Yellow arrows indicate articular cavities. Asterisks indicate the fat pad deep to the rectus femoris (RF), medial to the gluteus minimus (GMi), lateral to the iliopsoas (Ip), and superficial to the joint capsule (Cap). GMa = gluteus maximus, GMe = gluteus medius, Pe = pectineus, Sa = sartorius, TFL = tensor fasciae latae, *LT side* = lesser trochanter side, *GT side* = greater trochanter side.

Articular cavities and the fat pad in the inter-pericapsular muscle space showed postural differences among the three hip positions and in the joint capsule and pericapsular muscles (Fig. 4).

**Figure 5:**
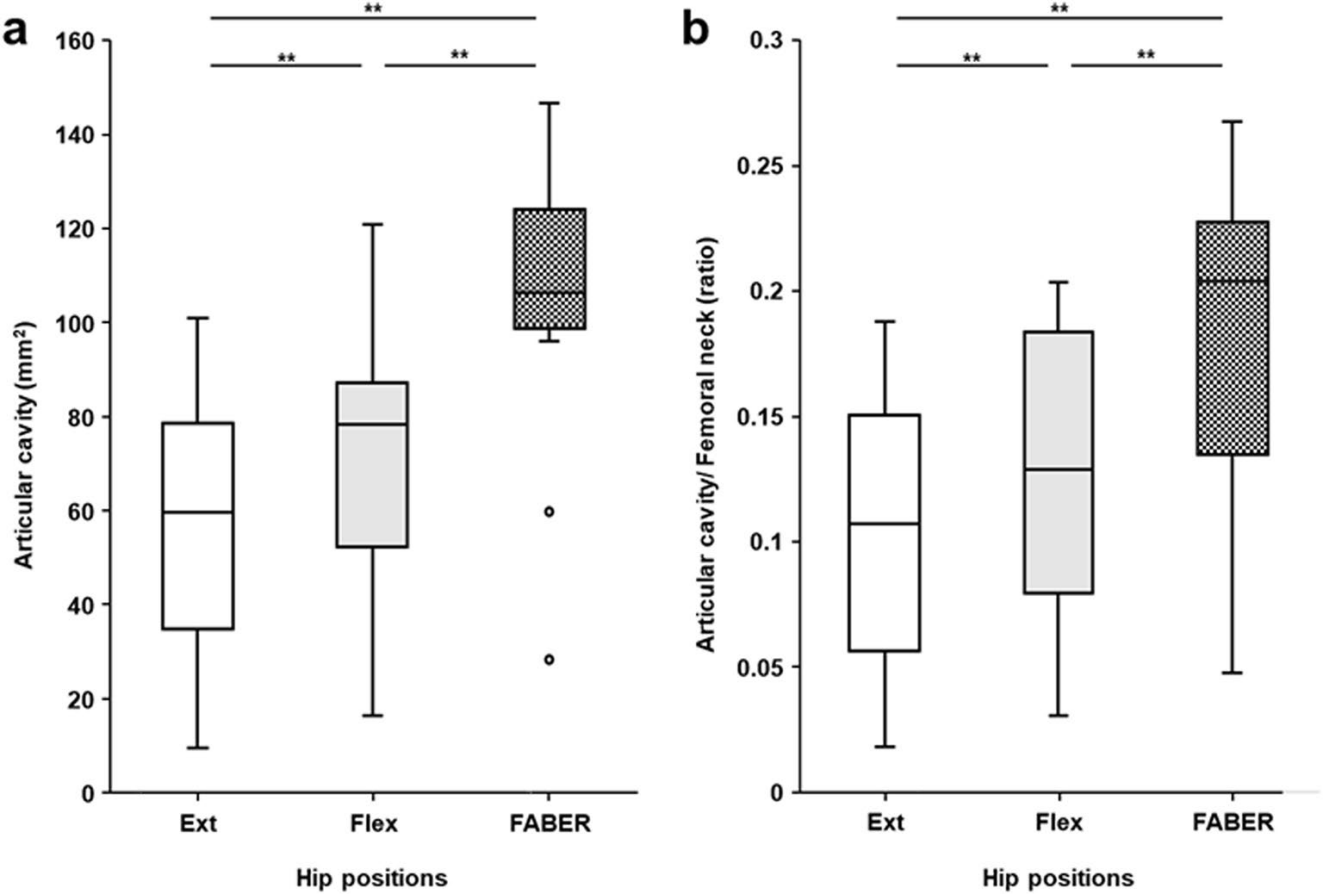
Comparison of the articular cavity area in three hip positions. Box plots show the area of the articular cavity (**a**) and the ratio of the articular cavity area to the narrowest area of the neck of femur (**b**) in the hip extension (Ext), hip flexion (Flex), and FABER positions. The height of the boxplot represents the interquartile range (IQR), and the black horizontal line inside the box represents the median. The lower and upper whiskers extend to the lowest and highest values within 1.5 IQR of the first and third quartiles, respectively. The circles represent outliers. The double asterisks (**) represent statistically significant differences (*p* < 0.017).

The area of the articular cavity was the largest in the FABER position (median, 106.5 mm^2^; IQR, 98.8–124.1 mm^2^), followed by the hip flexion (median, 78.4 mm^2^; IQR, 52.2–87.2 mm^2^) and extension (median, 59.7 mm^2^; IQR, 34.9–78.6 mm^2^) positions (Fig. 5a). Significant differences were observed in all comparisons between these areas in the three hip positions (FABER vs. hip flexion, *p* = 0.0015; FABER vs. hip extension, *p* = 0.0015; hip flexion vs. extension, *p* = 0.0015). The ratio of the area of the articular cavity to that of the neck of the femur was also the largest in the FABER position (median, 0.20; IQR, 0.13–0.23), followed by the hip flexion (median, 0.13; IQR, 0.08–0.18) and extension (median, 0.11; IQR, 0.06–0.15) positions (Fig. 5b). Significant differences were also observed in all comparisons between these ratios in the three hip positions (FABER vs. hip flexion, *p* = 0.0015; FABER vs. hip extension, *p* = 0.0015; hip flexion vs. extension, *p* = 0.0015).

**Figure 6:**
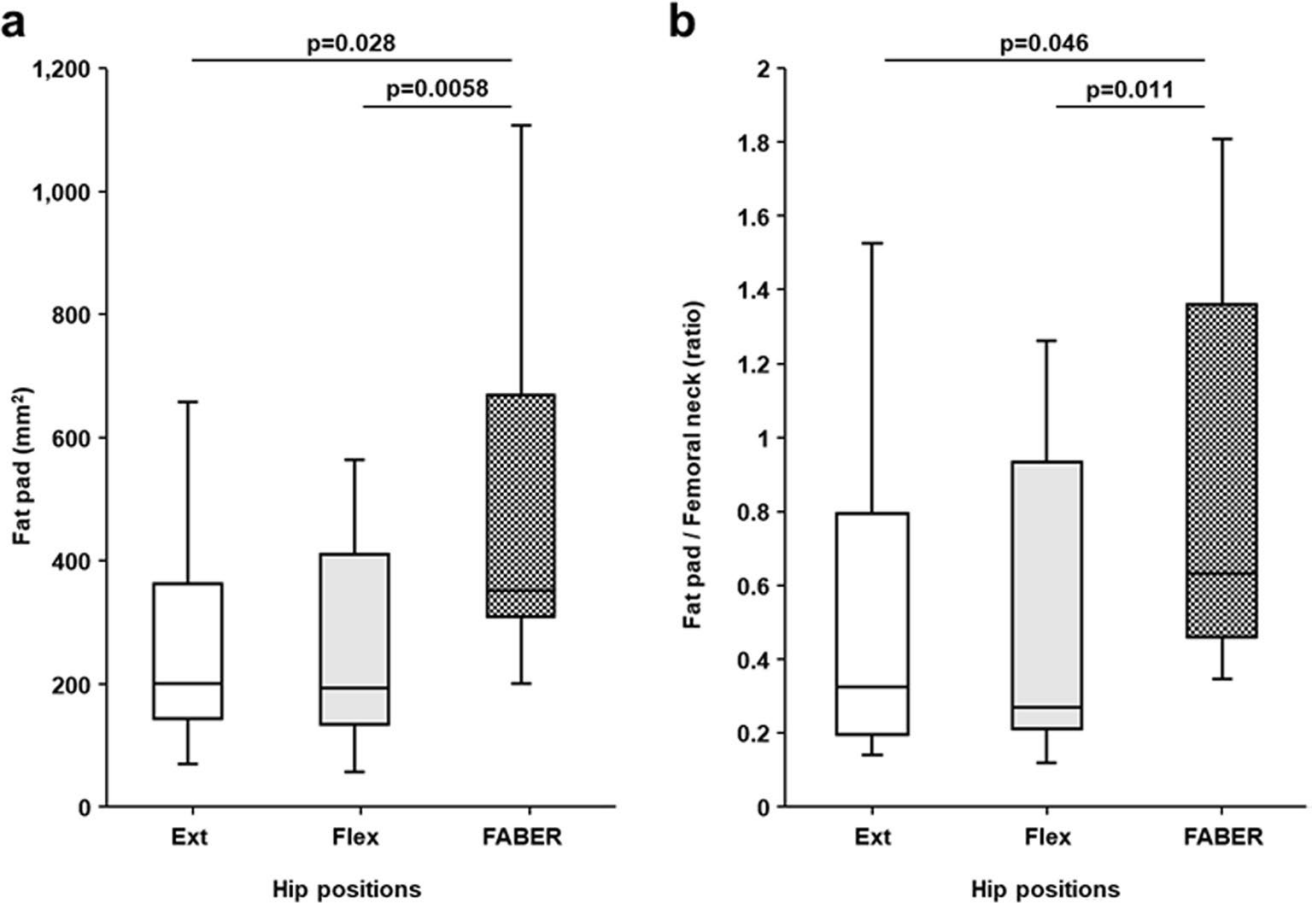
Comparison of the fat pad area in three hip positions. Box plots show the area of the fat pad (**a**) and its ratio to the narrowest area of the neck of femur (**b**) in the hip extension (Ext), hip flexion (Flex), and FABER positions. The height of the boxplot represents the interquartile range (IQR), and the black horizontal line inside the box represents the median. The lower and upper whiskers extend to the lowest and highest values within 1.5 IQR of the first and third quartiles, respectively.

The area of the fat pad in the FABER position (median, 352.5 mm^2^; IQR, 99.9–123.1 mm^2^) was significantly larger than those in hip flexion (median, 193.1 mm^2^; IQR, 133.9–410.1 mm^2^) (Fig. 6a, *p* = 0.0058). It was also larger than those in extension (median, 200.5 mm^2^; IQR, 144.1–363.4 mm^2^), though the difference was not statistically significant (*p* = 0.028). The ratio of the area of the fat pad to that of the femoral neck in the FABER position (median, 0.63; IQR, 0.46–1.36) was significantly larger than those in hip flexion (median, 0.27; IQR, 0.21–0.94) (Fig. 6b, *p* = 0.010). It was also larger than those in extension (median, 0.33; IQR, 0.20–0.80), though the difference was not statistically significant (*p* = 0.046).

## Discussion

The present study revealed that the area of the articular cavity and the ratio of the articular cavity area to the narrowest neck of the femur area in the FABER position were significantly larger than those in the hip flexion and extension positions. Additionally, the area of the fat pad in the inter-pericapsular muscle space and its ratio to the femoral neck in the FABER position were significantly larger than those in the hip flexion and, as a tendency, larger than those in hip extension.

Although some MR imaging studies have shown in vivo hip bone alignment at the FABER position^6,7^, joint capsule postural changes in the FABER position have been rarely discussed. The present study showed a significantly larger articular cavity area in the FABER position than in the hip flexion and extension positions. Based on cadaveric analysis using micro-computed tomography with a resolution of 200 µm, Tsutsumi et al. reported that the area of the articular cavity at the narrowest level of the neck of the femur can be used to evaluate the dynamic morphological change of the joint capsule as a whole^10^. Moreover, because the articular congruence of the hip joint increases during hip flexion, abduction, and external rotation, the joint capsule loosens in the FABER position^21,22^. Therefore, we obtained quantitative data proving that the FABER position loosens the joint capsule as a whole.

Several studies have highlighted the existence of the fat pad (of the inter-pericapsular muscle space) deep to the rectus femoris, medial to the gluteus minimus, lateral to the iliopsoas, and superficial to the joint capsule^12,23,24^ and suggested that its distribution may be significant in understanding the development of anterior hip pain^12^. The present study also confirmed the abovementioned finding regarding the fat pad and might be the first report to clarify its postural changes according to the hip position. Generally, the fat pad around the pericapsular muscles and joint capsule plays an essential role in promoting muscular force transmission and stress dissipation^25,26^. Therefore, we interpreted that the fat pad showed postural changes accompanied by pericapsular muscle and joint capsule postural changes.

Our findings indicated that in the healthy individuals, the joint capsule in the FABER position slackened, and the fat pad in the FABER position occupied larger region compared with those in other hip position as a result of the sufficient postural change of the joint capsule and pericapsular muscles. Recent anatomical studies have suggested that the joint capsule can undergo dynamic postural changes in relation to the pericapsular muscles^27–29^; therefore, this theoretical interrelationship between the joint capsule and pericapsular muscles, especially in the FABER position, may be supported by the findings of our in vivo study.

Our study had several limitations. First, the evaluation was limited to healthy individuals. Due to no comparison to pathological groups, we could not provide the clinically relevant information to be immediately applied in real clinical settings. Kaya indicated that some patients with FABER test-provoked anterior hip pain showed the pathological change on the fat pad superficial to the anterior joint capsule^11^. Based on our findings, future studies should evaluate whether the patients with hip pain also have postural changes of the joint capsule and fat pad in the FABER position to precisely understand the hip soft tissues which is subjected to mechanical stress in the FABER position. Second, imaging modality in our study (open MR) has disadvantage in terms of image resolution to create the three-dimensional image though the different hip positions can be easily imaged. Therefore, we could not perform the three-dimensional analysis of the femoral neck, joint capsule, pericapsular muscles, and fat pad. Third, we did not perform radiographic evaluation to avoid radiation exposure; thus, some osseous abnormalities could not be excluded. Fourth, because we only analyzed the left hips, we cannot eliminate any potential leg dominance effects on our findings. Finally, the sample size was relatively small, and only men were included.

In conclusion, the articular cavity area at the narrowest level of the neck of the femur in the FABER position was significantly larger than that in the hip flexion and extension positions. Moreover, the area of the fat pad in the inter-pericapsular muscle space in the FABER position were significantly larger than those in the hip flexion and, as a tendency, larger than those in hip extension.

## Supplementary Information

Supplementary Information.

## Data Availability

The datasets used and/or analyzed during the current study are available from the corresponding author upon reasonable request.
