# Supplementary material for: In vivo magnetic resonance imaging study of the hip joint capsule in the flexion abduction external rotation position
Source: Sci Rep. 2022 Apr 22;12:6656. doi: 10.1038/s41598-022-10718-7 (PMC9033789; doi:10.1038/s41598-022-10718-7)
Supplement: Supplementary file 1 — Supplementary Information. [file 41598_2022_10718_MOESM1_ESM.pdf]

***In vivo* Magnetic Resonance Imaging Study of the Hip Joint Capsule in the Flexion Abduction External Rotation Position**

Masahiro Tsutsumi, PhD[1, 2*], Isao Yamaguchi, PhD[3], Akimoto Nimura, MD, PhD[4], Hajime Utsunomiya, MD, PhD[5], Keiichi Akita, MD, PhD[2], and Shintarou Kudo, PhD[1]

[1] Inclusive Medical Science Research Institute, Morinomiya University of Medical Sciences, Osaka, JAPAN.

[2] Department of Clinical Anatomy, Graduate School of Medical and Dental Sciences, Tokyo Medical and Dental University, Tokyo, JAPAN.

[3] Department of Radiological Science, Faculty of Health Science, Morinomiya University of Medical Sciences, Osaka, JAPAN.

[4] Department of Functional Joint Anatomy, Graduate School of Medical and Dental Sciences, Tokyo Medical and Dental University, Tokyo, JAPAN.

[5] Tokyo Sports & Orthopaedic Clinic, Tokyo, JAPAN.

***Correspondence to:** Masahiro Tsutsumi, PhD, Inclusive Medical Science Research Institute, Morinomiya University of Medical Sciences, 1-26-16 Nankokita, Suminoe-ku,

Osaka city, Osaka, 559-8611, Japan

Phone: +81-6-6105-4090

Fax: +81-6-6616-6912

E-mail: masahiro_tsutsumi@morinomiya-u.ac.jp

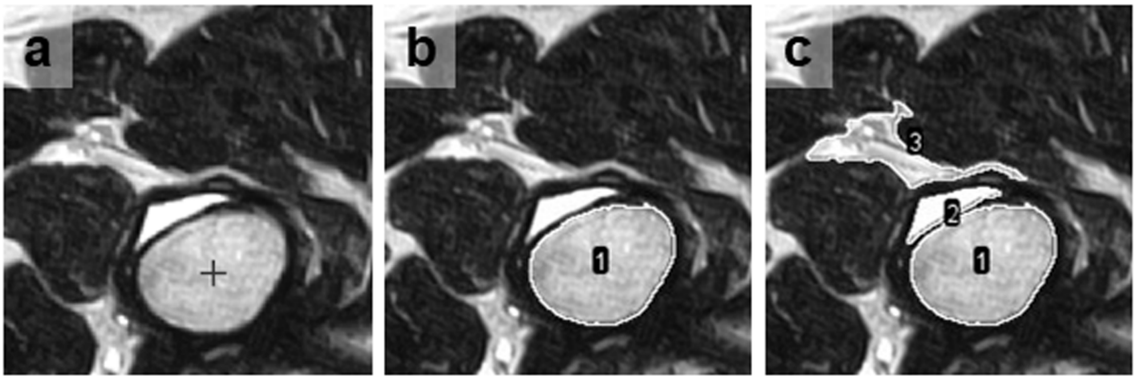

Supplementary Figure S1. Method of selecting each region of interest

Each region of interest was semi-automatically determined by “Wand tool” of ImageJ. By clicking inside the object (a: plus sign indicates the click region), the pixel value of the clicked area was extracted. Then, by setting the tolerance value, the outline of the object can be automatically selected under the condition that all pixel values in that area must be in the range “clicked pixel value − tolerance” to “clicked pixel value + tolerance” (b: the outline of the femoral neck can be semi-automatically determined by clicking inside it). Tolerance value was determined to ensure that no region beyond the outline of the object was selected. Using this method, all regions of interest can be selected (c: 1, femoral neck; 2, articular cavity; 3, fat pad).
